# Sex-Specific Clinical Outcomes After Treatment of Left Main Coronary Artery Disease. A NOBLE Substudy

**DOI:** 10.1016/j.jscai.2022.100338

**Published:** 2022-05-30

**Authors:** Margaret B. McEntegart, Niels R. Holm, Martin M. Lindsay, Keith G. Oldroyd, Timo Mäkikallio, David Hildick-Smith, Andrejs Erglis, Thomas Kellerth, Giedrius Davidavicius, Ian B.A. Menown, Lone J.H. Mogensen, Per H. Nielsen, Terje K. Steigen, Petter C. Endresen, Mark S. Spence, Alastair N.J. Graham, Peteris Stradins, Vesa Anttila, Leif Thuesen, Evald H. Christiansen, NOBLE Study Investigators

**Affiliations:** a Department of Cardiology, Golden Jubilee National Hospital, University of Glasgow, Glasgow, United Kingdom; b Department of Cardiology, Aarhus University Hospital, Skejby, Aarhus, Denmark; c Department of Cardiology, Oulu University Hospital, Oulu, Finland; d Sussex Cardiac Centre, Brighton and Sussex University Hospital, Brighton, United Kingdom; e Latvia Centre of Cardiology, Paul Stradins Clinical Hospital, Riga, Latvia; f Department of Cardiology, Örebro University Hospital, Örebro, Sweden; g Clinic of Cardiac and Vascular Disease, Institute of Clinical Medicine, Vilnius University, Vilnius, Lithuania; h Department of Cardiology, Craigavon Cardiac Centre, Craigavon, Northern Ireland; i Department of Cardiac Surgery, Aarhus University Hospital, Skejby, Aarhus, Denmark; j Cardiovascular Research Group, Department of Cardiology, UiT The Arctic University of Norway, University Hospital of North Norway, Tromsø, Norway; k Department of Cardiovascular Surgery, University Hospital of North Norway, Tromsø, Norway; l Belfast Heart Centre, Belfast Trust, Belfast, Northern Ireland; m Department of Thoracic Surgery, Belfast Heart Centre, Belfast Trust, Belfast, Northern Ireland; n Department of Thoracic Surgery, Latvia Centre of Cardiology, Paul Stradins Clinical Hospital, Riga, Latvia; o Department of Cardiac Surgery, Oulu University Hospital, Oulu, Finland; p Department of Cardiology, Aalborg University Hospital, Aalborg, Denmark

**Keywords:** Female, percutaneous coronary intervention, coronary artery bypass surgery, left main coronary artery disease

## Abstract

**Background:**

While female sex has been associated with worse outcomes following coronary revascularization, previous analyses in left main coronary artery (LMCA) disease have been conflicting. In addition, a signal that increased mortality may be specific to women treated with percutaneous coronary intervention (PCI) requires further investigation.

**Methods:**

Nordic-Baltic-British left main revascularization study (NOBLE) was a randomized trial comparing PCI to coronary artery bypass surgery (CABG) in patients with LMCA disease. The primary endpoint was a composite of all-cause mortality, nonprocedural myocardial infarction, repeat revascularization, and stroke (major adverse cardiovascular and cerebrovascular events [MACCE]). We report the 5-year sex-specific outcomes.

**Results:**

Of 1184 patients analyzed, 256 (22%) were female and 928 (78%) were male. There were no significant within-sex differences in baseline characteristics, disease location, or complexity between those treated with PCI and those with CABG. The 5-year MACCE rates were 29% and 15% in females and 28% and 20% in males treated with PCI and CABG, respectively. Within both sexes, there was an increased risk of MACCE with PCI compared with CABG, but no difference in all-cause mortality. On multivariate analysis, female sex was not an independent predictor of MACCE.

**Conclusions:**

Following the treatment of LMCA disease, long-term outcomes favored CABG over PCI in both sexes. Importantly, there was no difference in all-cause mortality in females or males at 5 ​years.

## Highlights

• There was no difference in 5-year mortality with CABG compared with PCI in both sexes.
• There was more MACCE at 5 years with PCI than CABG in both sexes.
• Female sex was not an independent predictor of 5-year MACCE.

## Central Illustration

**Figure:**
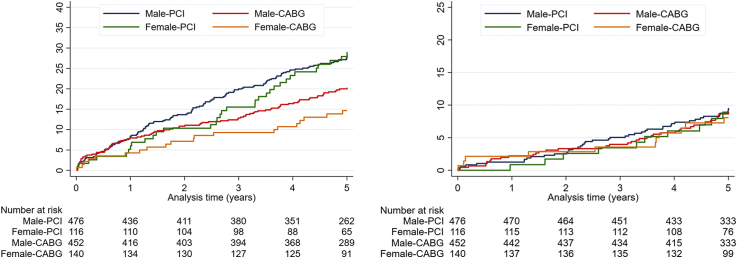

## Introduction

Until recent years, coronary artery bypass grafting (CABG) was the recommended treatment for left main coronary artery (LMCA) disease. Following publication of the Evaluation of XIENCE Versus Coronary Artery Bypass Surgery for Effectiveness of Left Main Revascularization (EXCEL) and Nordic-Baltic-British left main revascularization (NOBLE) multicenter international, randomized, controlled trials^1^^,^^2^ and subsequent meta-analyses,^3^, ^4^, ^5^ percutaneous coronary intervention (PCI) has been increasingly adopted in the treatment of LMCA disease.

While the Synergy between Percutaneous Coronary Intervention with Taxus and Cardiac Surgery (SYNTAX) trial was a randomized comparison of PCI vs CABG in patients with multivessel disease (N = 1800), 40% of the patients had LMCA disease. While CABG outcomes were comparable between women and men, female sex was an independent predictor of long-term mortality in the PCI cohort, and thus was included in the SYNTAX II score, a tool to guide revascularization strategy.^6^, ^7^, ^8^

The EXCEL trial (N = 1905) reported noninferiority for PCI compared with CABG in patients with LMCA disease and low to intermediate SYNTAX scores at 5 ​years of follow-up.^9^ After multivariate analysis, female sex was not an independent predictor of the composite primary endpoint of all-cause death, myocardial infarction (MI), or stroke or for all-cause mortality alone at 3 ​years.^10^

While the NOBLE trial (N = 1201), which also compared PCI to CABG in patients with LMCA disease, found no difference in all-cause mortality, the composite primary endpoint of mortality, nonprocedural MI, repeat revascularization, and stroke (major adverse cardiac and cerebrovascular events [MACCE]) was higher at 5 ​years in patients treated with PCI.^11^ In this analysis, we describe the baseline characteristics and long-term clinical outcomes for female and male patients treated with PCI or CABG in the NOBLE trial.

## Methods

The NOBLE study was a multicenter international, prospective, open-label, randomized, noninferiority trial comparing PCI to CABG in patients with LMCA disease (ISRCTN87206264; ClinicalTrials.gov identifier: NCT01496651). The trial design, methods, and results have previously been reported.^2^ The key inclusion criteria for enrolment were stable angina or unstable angina/acute coronary syndrome, with a LMCA lesion visually assessed as ≥50% stenosis or fractional flow reserve ≤0.80 in the ostium, mid-shaft, or bifurcation and no more than 3 additional noncomplex lesions.

The primary endpoint was a composite of MACCE (death from any cause, nonprocedural MI, repeat revascularization, or stroke) at median 3-year follow-up. Details of all trial endpoints and definitions have previously been described.^2^

In this analysis, baseline demographics, clinical characteristics and presentation, disease location and complexity, and 5-year clinical outcomes are compared for the 2 treatment strategies in female and male patients.

### Statistical analysis

Continuous variables are reported as mean (± standard deviation) and compared using *t* tests if normally distributed. Non-normalized data are reported as median [interquartile range] and compared using the Mann-Whitney *U* test. Categorical variables are reported as counts (percentage, %), and differences between groups were assessed with the χ^2^ test. A 2-sided *P*-value of less than 0.05 was considered significant. Clinical event rates are presented using Kaplan–Meier curves, and groups were compared using the log-rank test. Forest plots present hazard ratio (HR) by unadjusted Cox-regression analysis with 95% confidence intervals (CI). The assumption of proportional hazards in the Cox-regression model was assessed graphically by a plot of observed vs predicted events and by log-log plot. The assumptions were fulfilled except for the endpoint of stroke. All analyses were performed using Stata 15 (StataCorp).

## Results

Between December 9, 2008, and January 21, 2015, 1201 patients were enrolled from 36 centers in northern Europe. Fourteen patients withdrew consent, 3 were lost to follow-up, and 1184 were included in the analysis with follow-up for 5 ​years.

Of the 1184 patients, 22% were female (n = 256), and 78% male (n = 928). Of the female patients, 45% were treated with PCI (n = 116), and 55% with CABG (n = 140). Of the male patients, 51% were treated with PCI (n = 476), and 49% with CABG (n = 452).

In comparison to males, females had a higher prevalence of diabetes, hypertension, and statin therapy, but less often had previous PCI, distal LMCA disease, and a lower mean SYNTAX score (Table 1).

**Table 1:** Baseline characteristics.

	Female PCI (n = 116)	Female CABG (n = 140)	Female overall (n = 256)	Male PCI (n = 476)	Male CABG (n = 452)	Male overall (n = 928)	*P* value female vs male
Age, y	67 ​± ​9	67 ​± ​10	67 ​± ​10	66 ​± ​10	66 ​± ​9	66 ​± ​10	.10
Body mass index, kg/m^2^	28 ​± ​5	29 ​± ​5	29 ​± ​5	28 ​± ​4	28 ​± ​4	28 ​± ​4	.02
Diabetes type I or type II	21 (18%)	32 (23%)	53 (21%)	69 (15%)	62 (14%)	131 (14%)	.01
Family history of IHD	70 (63%)	87 (66%)	157 (65%)	251 (57%)	220 (53%)	471 (55%)	.006
Statin treatment	97 (84%)	120 (86%)	217 (85%)	385 (81%)	344 (76%)	729 (79%)	.03
Hypertension	84 (72%)	98 (70%)	182 (71%)	302 (64%)	291 (64%)	593 (64%)	.04
Active smoking	21 (18%)	25 (18%)	46 (18%)	87 (19%)	102 (23%)	189 (21%)	.41
Previous PCI	13 (11%)	22 (16%)	35 (14%)	103 (22%)	96 (21%)	199 (22%)	.006
Previous CABG	1 (0.7%)	1 (0.9%)	2 (0.8%)	3 (0.6%)	1 (0.2%)	4 (0.4%)	.62
Ejection fraction, %	60 [55-65]	60 [55-65]	60 [55-65]	60 [55-65]	60 [50-62]	60 [52-63]	.005
NYHA class							
I	46 (51%)	31 (33%)	77 (42%)	198 (54%)	164 (45%)	362 (50%)	
II	23 (26%)	38 (40%)	61 (33%)	112 (30%)	112 (31%)	224 (31%)	
III	14 (16%)	18 (19%)	32 (17%)	43 (12%)	59 (16%)	102 (14%)	
IV	7 (7%)	7(8%)	14 (8%)	16 (4%)	26 (7%)	42 (6%)	.25
EUROSCORE	2.5 [1-4]	3 [2-4]	3 [2-4]	2 [1-4]	2 [1-4]	2 [1-4]	<.0001
SYNTAX score	22.0 ​± ​7.9	20.6 ​± ​7.4	21.3 ​± ​7.6	22.5 ​± ​7.3	22.8 ​± ​7.9	22.7 ​± ​7.6	.009
Indication							
Stable angina pectoris	89 (77%)	114 (82%)	203 (80%)	397 (83%)	377 (83%)	774 (83%)	.16
Unstable angina pectoris	27 (23%)	25 (18%)	52 (20%)	79 (17%)	75 (17%)	154 (17%)	.16
Lesions to be treated, n	2 [1-3]	2 [2-3]	2 [1-3]	2 [1-3]	2 [2-3]	2 [1-3]	.85
Distal LMCA lesion	87 (75%)	106 (76%)	193 (75%)	390 (82%)	376 (83%)	766 (83%)	.01
Balloon/stent size, mm	4 [3.5-4.5]			4 [4-5]			<.001

Values are mean ± standard deviation, n (%), or median [interquartile range].

CABG, coronary artery bypass grafting; IHD, ischemic heart disease; IQR, interquartile range; LMCA, left main coronary artery; NYHA, New York Heart Association; PCI, percutaneous coronary intervention; SYNTAX, Synergy Between Percutaneous Coronary Intervention With Taxus and Cardiac Surgery.

Within both sexes, there was no significant difference in the baseline demographics, clinical presentation, disease complexity scores, or prevalence of distal LMCA disease, in those treated with PCI vs CABG. In both sexes, more lesions were intended to be treated with CABG than with PCI.

During PCI, the largest balloon or stent used in the LMCA was smaller in females than in males (mean diameter 4.1 ​± ​0.6 vs 4.3 ​± ​0.6 ​mm; *P* < .001), while there was no difference in the maximum pressure used to deploy this device (mean pressure females 17.6 ​± ​3.6 vs males 17.7 ​± ​4.1 ​atm; *P* = .92). There was no difference in the number of stents used to treat the LMCA disease (females 1.5 ​± ​0.8 stents vs males 1.5 ​± ​0.7; *P* = .34). Although the difference was not significant, intravascular ultrasound (IVUS) was used less often in females than in males both before (44% vs 46%; *P* = .69) and after PCI (66% vs 74%; *P* = .09).

**Figure 1:**
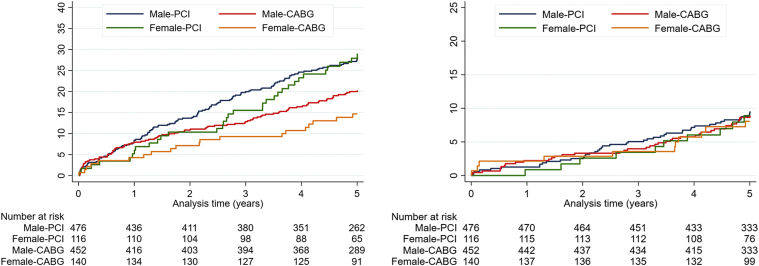
Kaplan-Meier curves, major adverse cardiac and cerebrovascular events and death.

**Figure 2:**
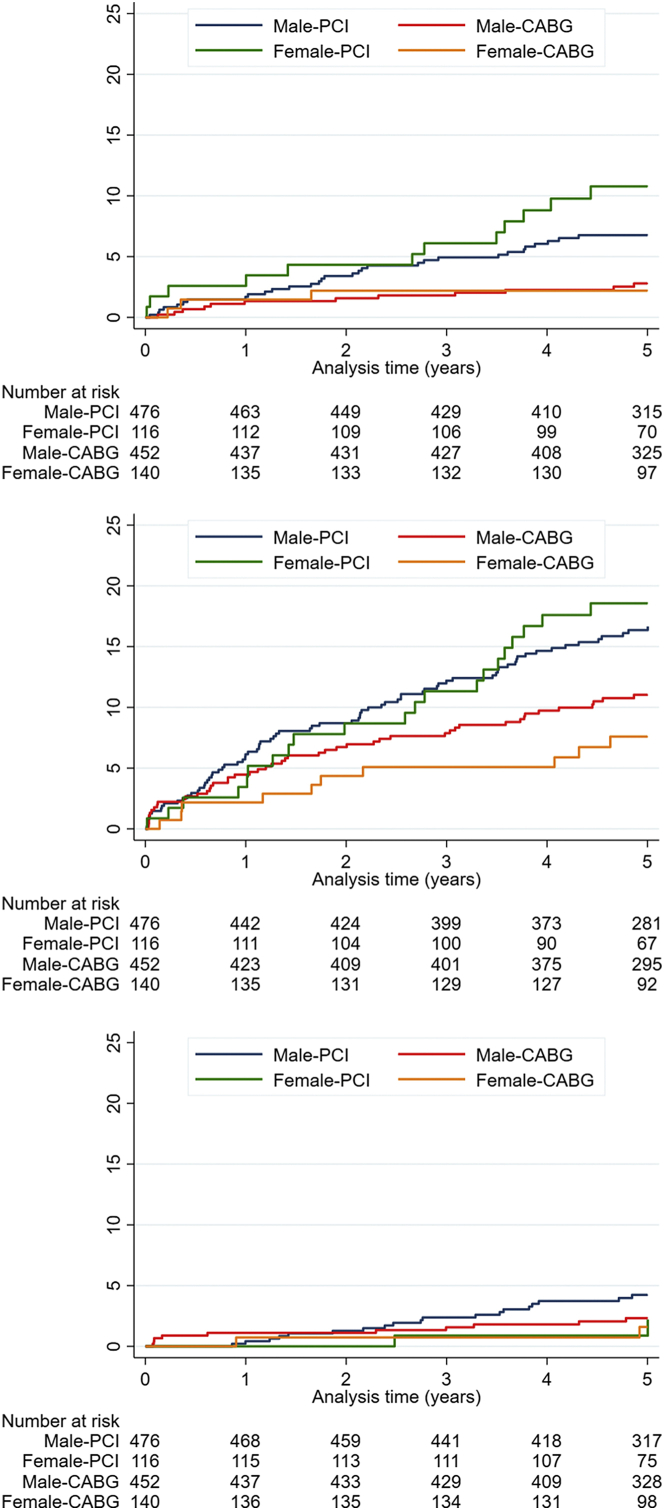
Kaplan-Meier curves, individual components of MACCE.

The 5-year MACCE rates were 29% and 15% in females and 28% and 20% in males treated with PCI and CABG, respectively (log-rank, *P* = .001) (Figure 1, Central Illustration and Figure 1, Central Illustration). The event rates for the individual clinical components of MACCE are reported in Table 2 and illustrated in Figure 2.

**Central Illustration:**
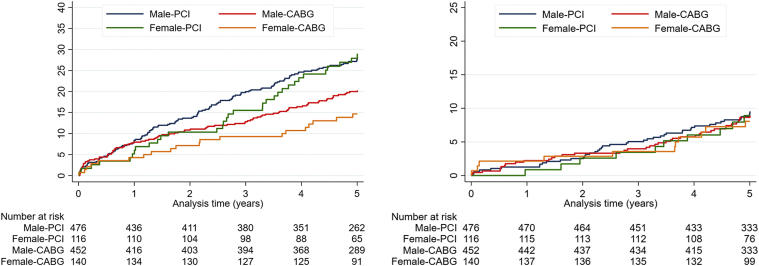
Five-year major adverse cardiovascular and cerebrovascular events and mortality according to sex and revascularization.

**Table 2:** Kaplan-Meier 5-year estimates.

Endpoint	Female (n = 256)				Male (n = 928)			
	PCI KM 5-y estimate (n = 116)	CABG KM 5-y estimate (n = 140)	HR (95% CI)	*P* value	PCI KM 5-y estimate (n = 476)	CABG KM 5-y estimate (n = 452)	HR (95% CI)	*P* value
MACCE	29% (33)	15% (20)	2.12 (1.21-3.69)	.007	28% (132)	20% (90)	1.46 (1.11-1.90)	.006
All-cause mortality	9% (10)	8% (11)	1.09 (0.46-2.58)	.84	10% (44)	9% (39)	1.08 (0.70-1.66)	.74
Nonprocedural myocardial infarction	11% (12)	2% (3)	4.94 (1.40-17.52)	.006	7% (31)	3% (12)	2.50 (1.29-4.87)	.005
Repeat revascularization	19% (21)	8% (10)	2.66 (1.25-5.64)	.008	17% (76)	11% (48)	1.54 (1.07-2.21)	.02
Stroke	0.02% (2)	0.01% (2)	NA	.67	4% (19)	2% (10)	1.82 (0.85-3.92)	.12

Values are % (n) unless otherwise noted.

CABG, coronary artery bypass grafting; CI, confidence interval; HR, hazard ratio; KM, Kaplan-Meier; MACCE, major adverse cardiovascular and cerebrovascular events; PCI, percutaneous coronary intervention.

**Figure 3:**
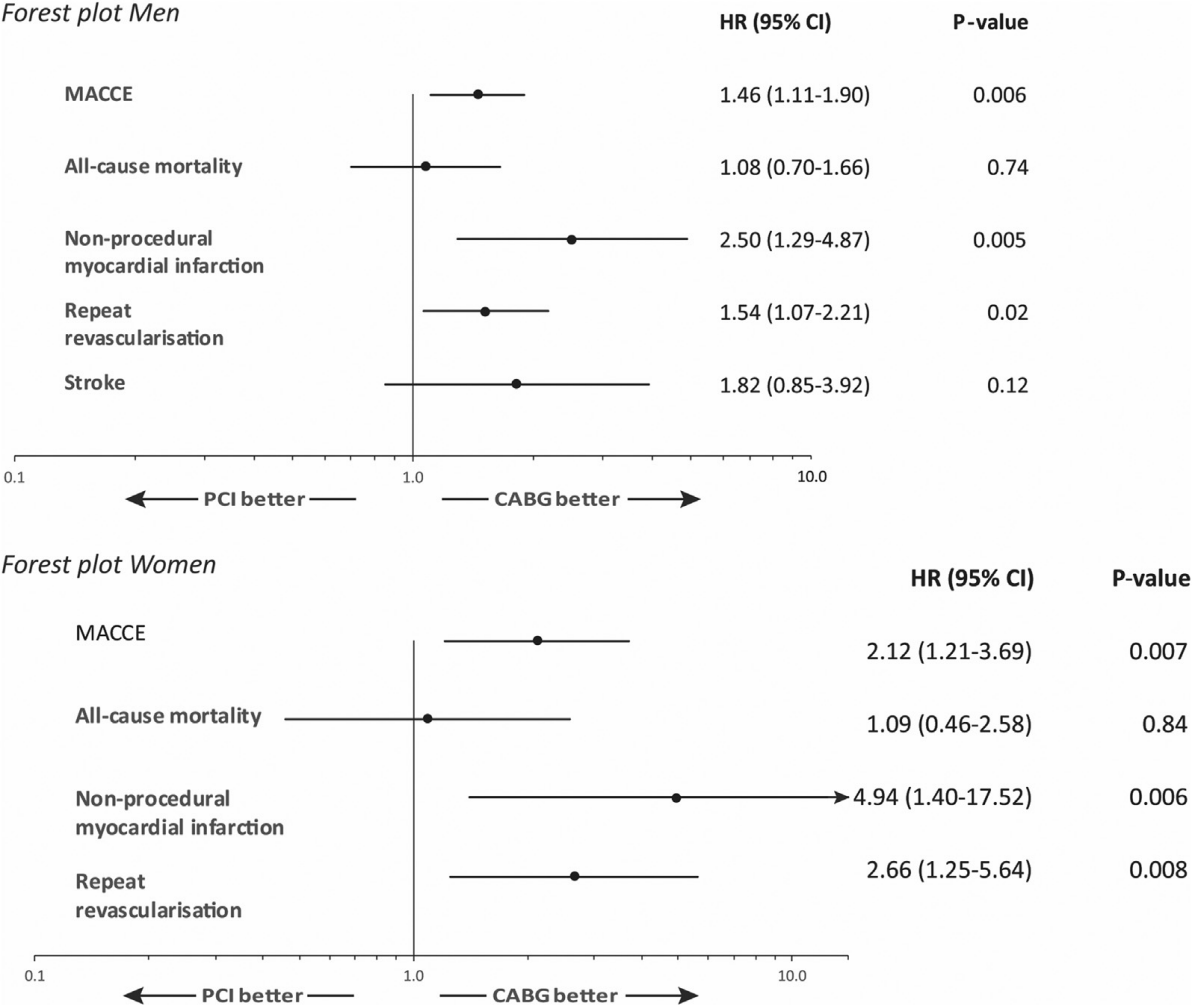
Forest plots, PCI vs CABG.

Within both sexes, there was an increased risk of MACCE with PCI compared with CABG (females HR, 2.12; 95% CI, 1.21-3.69; *P* = .007; males HR, 1.46; 95% CI, 1.11-1.90; *P* = .006) (Table 3) (Figure 3). This was driven in both sexes by an increased hazard for nonprocedural MI and repeat revascularization, while there was no increased risk for all-cause mortality.

**Table 3:** Multivariable analyses for MACCE composite endpoint and nonprocedural myocardial infarction.

	HR (95% CI)	*P* value
MACCE composite endpoint		
PCI (vs CABG)	1.58 (1.24-2.00)	<.0001
Age (per year)	1.03 (1.01-1.04)	<.0001
Diabetes (vs no diabetes)	1.64 (1.23-2.19)	.001
SYNTAX score (per 1 unit)	1.02 (1.00-1.04)	.014
Female (vs men)	0.81 (0.60-1.09)	.17
Nonprocedural myocardial infarction		
PCI (vs CABG)	2.95 (1.64-5.31)	<.0001
Age (per year)	1.04 (1.01-1.08)	.004
SYNTAX score (per 1 unit)	1.04 (1.01-1.07)	.017
Female (vs men)	1.30 (0.72-2.35)	.38

CABG, coronary artery bypass grafting; CI, confidence interval; HR, hazard ratio; MACCE, major adverse cardiovascular and cerebrovascular events; PCI, percutaneous coronary intervention; SYNTAX, Synergy Between Percutaneous Coronary Intervention With Taxus and Cardiac Surgery.

The independent predictors of 5-year MACCE rates were analyzed in a multivariate Cox proportional hazard model. Only the significant variables were retained in the final model, with sex added later. In addition, the interaction between sex and treatment and between sex and age were tested in the same model without giving anything further to the result. While PCI, age, diabetes, and SYNTAX score were significant variables, female sex was not an independent predictor of long-term MACCE (Table 3). A further analysis of predictors of 5-year nonprocedural MI (the endpoint component associated with most hazard) found PCI, age, and SYNTAX score, but not female sex, to be independent predictors of this outcome.

## Discussion

The key findings in this study assessing sex-specific long-term outcomes after coronary revascularization in patients with LMCA disease are that (1) in both females and males, the composite primary endpoint of MACCE favored CABG over PCI, with a stronger treatment effect in females; (2) in both sexes, there was no difference in all-cause mortality between those treated with PCI and those with CABG; (3) females had a higher prevalence of cardiovascular risk factors but a less complex pattern of coronary artery disease; (4) female sex was not an independent predictor of long-term outcomes.

The SYNTAX trial substudy of patients with LMCA and 3-vessel disease found that females treated with PCI compared with CABG had higher all-cause mortality at 4 ​years.^6^ The EXCEL trial also found that mortality tended to be higher in females treated with PCI but, in contrast to SYNTAX, the difference was not significant compared with females treated with CABG or males treated with PCI or CABG.^10^ In addition, and consistent with our findings, in EXCEL, female sex was not an independent predictor of mortality or the composite primary endpoint at 3 ​years.^1^^,^^10^

In our analysis, the stronger treatment effect in female patients was driven by an almost 5-fold increase in hazard for nonprocedural MI and 2/3-fold increased risk of repeat revascularization. In EXCEL, the composite of procedural and nonprocedural MI trended higher in females (11.7% vs 6.8%; *P* = .08) and lower in males (6.9% vs 8.8%; *P* = .12) after PCI than after CABG at 3 ​years. In our analysis, nonprocedural MI was significantly higher in both females (10.8% vs 2.2%) and males (6.8% vs 2.8%) treated with PCI than in those treated with CABG at 5 ​years. In EXCEL, ischemia-driven revascularization trended higher in females (14.1% vs 9.8%; *P* = .17) and was significantly higher in males (12.1% vs 6.8%; *P* = .001) after PCI than after CABG at 3 ​years. In our analysis, repeat revascularization was significantly higher in both females (18.6% vs 7.6%) and males (16.7% vs 11.0%) treated with PCI than with CABG at 5 ​years.

There are several possible contributing mechanistic explanations for these observations to consider. First, females had a higher prevalence of diabetes, hypertension, and hyperlipidemia, as was also observed in EXCEL. In addition, EXCEL also reported a higher incidence of procedural ischemic and bleeding complications in female patients, which are known to be associated with worse long-term clinical outcomes.^10^ Another pathophysiological sex difference to consider is the higher incidence of MI with nonobstructive coronary arteries in female patients, which could have contributed to the higher rate of nonprocedural MI.^12^

An important anatomical sex difference to consider is that smaller caliber coronary arteries in female patients result in smaller minimal stent areas (MSA),^13^ which in the LMCA has been associated with poorer clinical outcomes.^14^ In this analysis, we found that the largest balloon or stent used to treat the LMCA disease was significantly smaller in female patients than in male patients. In our NOBLE IVUS substudy, patients were divided into 3 groups according to LMCA MSA, with the lowest tertile associated with a significantly higher rate of repeat revascularization and LMCA target lesion revascularization.^15^ In the EXCEL IVUS substudy, female sex was associated with a smaller vessel size and MSA.^16^ Of note, while we found the use of IVUS was lower in females, in EXCEL, IVUS use was lower in males.^1^

Lastly, while previous studies have reported sex differences in guideline-directed medical therapy, EXCEL reported no difference including dual antiplatelet therapy at 3 ​years.

Further data are required to determine whether, in the context of LMCA disease, our threshold for CABG in female and male patients should be the same.

### Study limitations

The main limitation of this study is that it is a subgroup analysis and thus should be considered as hypothesis generating.

## Conclusion

Long-term composite clinical outcomes favored CABG over PCI in the treatment of LMCA disease in both sexes, but there was no difference in all-cause mortality in female or male patients at 5 ​years. Female sex was not an independent predictor of 5-year MACCE.

## Declaration of competing interest

Dr McEntegart has received consultancy fees from Abbott Vascular and Boston Scientific. Dr Holm has received institutional research grants from 10.13039/501100008877Biosensors, 10.13039/100001316Abbott, 10.13039/100015305Reva Medical, Medis Medical Imaging, and 10.13039/100008497Boston Scientific and speaker fees from Terumo, Abbott, Reva Medical, and Medis Medical Imaging. Dr Oldroyd has received speaker fees from Biosensors and Abbott Vascular. Dr Erglis has received institutional research grants from 10.13039/100011949Abbott Vascular and 10.13039/100008497Boston Scientific and consultancy fees from Abbott Vascular, Biosensors, Boston Scientific, Cordis J&J, and Medtronic. Dr Christiansen has received grants from 10.13039/501100008877Biosensors. None of the other authors have any conflicts of interest.
